# School-Based Fluoride Mouth-Rinse Program Dissemination Associated With Decreasing Dental Caries Inequalities Between Japanese Prefectures: An Ecological Study

**DOI:** 10.2188/jea.JE20150255

**Published:** 2016-11-05

**Authors:** Yusuke Matsuyama, Jun Aida, Katsuhiko Taura, Kazunari Kimoto, Yuichi Ando, Hitoshi Aoyama, Manabu Morita, Kanade Ito, Shihoko Koyama, Akihiro Hase, Toru Tsuboya, Ken Osaka

**Affiliations:** 1 Department of International and Community Oral Health, Tohoku University Graduate School of Dentistry, Sendai, Japan; 1 東北大学大学院歯学研究科 国際歯科保健学分野、仙台、日本; 2 Non-profit Japanese Conference on the Promotion of the Use of Fluoride in Caries Prevention, Mizuho, Gifu, Japan; 2 NPO法人日本フッ化物むし歯予防協会、瑞穂、岐阜、日本; 3 Division of Oral Health, Department of Oral Science, Graduate School of Dentistry, Kanagawa Dental University, Yokosuka, Kanagawa, Japan; 3 神奈川歯科大学大学院歯学研究科 口腔科学講座 口腔衛生学分野、横須賀、神奈川、日本; 4 National Institute of Public Health, Wako, Saitama, Japan; 4 国立保健医療科学院、生涯健康研究部、和光、埼玉、日本; 5 Tochigi Prefectural Medical and Welfare College, Utsunomiya, Japan; 5 栃木県立衛生福祉大学校歯科技術学部、宇都宮、日本; 6 Department of Preventive Dentistry, Okayama University Graduate School of Medicine, Dentistry and Pharmaceutical Sciences, Okayama, Japan; 6 岡山大学大学院医歯薬学総合研究科予防歯科学分野、岡山、日本; 7 Division of Oral Health Sciences, Department of Health Sciences, School of Health and Social Services, Saitama Prefectural University, Koshigaya, Saitama, Japan; 7 埼玉県立大学 保健医療福祉学部 健康開発学科 口腔保健科学専攻、越谷、埼玉、日本; 8 Department of Social and Behavioral Sciences, Harvard T.H. Chan School of Public Health, Boston, MA, USA; 8 ハーバード・チャン公衆衛生大学院 社会行動科学学部 ボストン、マサチューセッツ、米国

**Keywords:** fluoride, dental caries, population approach, health inequalities

## Abstract

**Background:**

Dental caries inequalities still severely burden individuals’ and society’s health, even in countries where fluoride toothpastes are widely used and the incidence of dental caries has been decreasing. School-based fluoride mouth-rinse (S-FMR) programs, a population strategy for dental caries prevention, might decrease dental caries inequalities. This study investigated the association between S-FMR and decreasing dental caries prevalence and caries-related inequalities in 12-year-olds by Japanese prefecture.

**Methods:**

We conducted an ecological study using multi-year prefecture-level aggregated data of children born between 1994 and 2000 in all 47 Japanese prefectures. Using two-level linear regression analyses (birth year nested within prefecture), the association between S-FMR utilization in each prefecture and 12-year-olds’ decayed, missing, or filled permanent teeth (DMFT), which indicates dental caries experience in their permanent teeth, were examined. Variables that could explain DMFT inequalities between prefectures, such as dental caries experience at age 3 years, dentist density, and prefectural socioeconomic circumstances, were also considered.

**Results:**

High S-FMR utilization was significantly associated with low DMFT at age 12 (coefficient −0.011; 95% confidence interval, −0.018 to −0.005). S-FMR utilization explained 25.2% of the DMFT variance between prefectures after considering other variables. Interaction between S-FMR and dental caries experience at age 3 years showed that S-FMR was significantly more effective in prefectures where the 3-year-olds had high levels of dental caries experience.

**Conclusions:**

S-FMR, administered to children of all socioeconomic statuses, was associated with lower DMFT. Utilization of S-FMR reduced dental caries inequalities via proportionate universalism.

## Abstract (ja)

**背景：:**

フッ化物配合歯磨剤が普及し、むし歯（う蝕）が減少している国でも、むし歯の健康格差は個人や社会の大きな負担となっている。保育園・幼稚園・小中学校・特別支援学校で実施される“学校フッ化物洗口（集団フッ化物洗口）”は、むし歯予防の優れたポピュレーションアプローチであり、むし歯の健康格差を縮小させている可能性がある。本研究は日本における学校フッ化物洗口と12歳児1人平均永久歯むし歯経験歯数（Decayed, Missing, or Filled permanent Teeth Index, DMFT指数）の関連およびその都道府県格差との関連を明らかにすることを目的とした。

**方法：:**

1994–2000年に生まれた子どもに関する、複数年度の都道府県レベルの集計データを使用し、地域相関研究をした。都道府県をレベル2、出生年度をレベル1とした2レベルのマルチレベル線形回帰分析により、学校フッ化物洗口を受けている児童の割合（学校フッ化物洗口普及割合）と12歳児DMFT指数（平均永久歯むし歯経験歯数）の関連を検討した。共変量は3歳児1人平均乳歯むし歯経験歯数、歯科医師密度、都道府県の社会経済状況などとした。

**結果：:**

都道府県別の学校フッ化物洗口普及割合が高いことと12歳児DMFT指数が小さいことは有意に関連していた（B = -0.011; 95%信頼区間, -0.018 to -0.005）。他の変数を考慮してもなお、学校フッ化物洗口普及割合は12歳児DMFT指数の都道府県レベル分散を25.2%説明した。学校フッ化物洗口普及割合と3歳児乳歯むし歯経験歯数の間に有意な交互作用がみられ、学校フッ化物洗口普及割合と12歳児DMFT指数の負の関連は、3歳児乳歯むし歯経験歯数が多い都道府県で大きかった。

**結論：:**

学校フッ化物洗口普及割合が高いことは、12歳児DMFT指数が小さいことと有意に関連していた。子どもの家庭環境や社会経済状況によらずに予防効果を受けることができる学校フッ化物洗口は、比例的普遍アプローチとしてはたらき、むし歯の健康格差を縮小していた。

## INTRODUCTION

International and domestic health inequalities have emerged as important research topics^1^ and represent a global public health issue.^1^^–^^3^ Dental caries was the most common disease in the Global Burden of Disease 2010 Study,^4^ and its global inequalities are remarkable.^5^^–^^9^ Even in developed countries, there are significant variations in dental health inequality by area.^10^^,^^11^ Owing to high dental disease prevalence, the total cost of medical care for dental diseases is the highest across all diseases in Japan, exceeding 26 billion United States dollars (1 US dollar ≈ 100 Japanese yen) in 2011.^12^ Thus, despite the recent decline in dental caries, the health burden of dental caries on individuals and societies remains high.

Health intervention dissemination is often affected by socioeconomic circumstances.^13^ Therefore, some interventions do not necessarily reduce health inequalities even if their health efficacy is established by randomized controlled trials. In fact, interventions that depend on individual motivation often increase health inequalities.^13^^,^^14^ In contrast, interventions aimed at changing social environment are beneficial regardless of individual socioeconomic circumstances and can reduce health inequalities. They are sometimes more beneficial for people with poor socioeconomic circumstances.^3^^,^^15^^,^^16^ Such interventions are known as population strategies.^17^ Water fluoridation is an example of a population strategy^16^^,^^17^ that reduces dental health inequalities.^15^^,^^18^ Unfortunately, water fluoridation has not been established in Japan except on United States Army bases, even though it is recommended by Japanese Society for Oral Health^19^ and Ministry of Health, Labour and Welfare stated for technical support to fluoridation.^20^ Japanese government and advocate activities are conducted in several municipalities.

Some schools in Japan have adopted another population strategy for dental caries prevention, namely a school-based fluoride mouth-rinse (S-FMR) programs. S-FMR offers the possibility of decreasing dental caries inequalities among schoolchildren.^21^ The Japanese government has published guidelines for fluoride (FMR) mouth-rinse.^22^ However, the proportion of schoolchildren receiving S-FMR differs among prefectures^23^ because some prefectures have not included S-FMR as part of the local public health policy, and the final decision to introduce S-FMR is made by each school’s administrators. S-FMR works as a “geographical targeting population approach,” a population approach for specific areas.^24^^,^^25^ Thus, it might partially contribute to children’s dental health improvement and decrease inequalities between prefectures in Japan.

S-FMR changes dental caries prevention strategies at the school level. It improves schoolchildren’s dental health regardless of their socioeconomic status, and is more effective for schoolchildren with poor dental status.^21^^,^^26^ In contrast, using fluoride toothpaste at home depends on individual household efforts and may thus be affected by social determinants.^27^ A recent Cochrane systematic review showed that simultaneously receiving FMR and using fluoride toothpaste is more effective than the use of fluoride toothpaste alone, but not to a significant degree.^28^ However, another review showed that the use of a topical fluoride application, including mouth-rinse, significantly reduced dental caries.^29^ Thus, the effectiveness of S-FMR is questionable in countries like Japan, where fluoride toothpaste is widely used.

Here, we examined the effectiveness S-FMR on children’s dental caries experience and inequalities at the population level between prefectures in Japan, accounting for prefectural differences in socioeconomic circumstances, fluoride toothpaste utilization, and the recent decline in dental caries.

## METHODS

### Study design

To examine S-FMR effectiveness in decreasing children’s dental caries experience at a population level and dental caries inequalities between prefectures, we chose an ecological study design using each prefecture as one unit. Ecological studies are frequently used to investigate the spatial patterns of diseases and interventions.^30^ Ecological study is appropriate when the prevention or intervention implications are at a population level.^31^ This study design is sometimes criticized for weak causal inference, since associations observed on a population level are not necessarily applicable on an individual level. However, this “ecological fallacy” can be avoided when causation has been shown in previous studies.^32^ Causal relationships between the use of fluoride mouth-rinse and caries prevention have been established in randomized controlled trials.^29^ Thus, we believe that the ecological study design has greater benefits than disadvantages for our research.

In addition, to consider recent declines in dental caries and high utilization of fluoride toothpaste in Japan, we used data from multi-year birth cohorts, aggregated data to the prefecture level, and employed multilevel analysis. We obtained the prefecture-level aggregated variables of all 47 prefectures in Japan relating to children born between 1994 and 2000 from open data from previous surveys conducted when the children were aged 3, 7, and 12 years. The variables were grouped according to the age of each birth cohort (eFigure 1 and eTable 1). Thus, the data set included the data of 329 units (seven birth cohorts in 47 prefectures).

### Dental caries status at ages 3 and 12 years

Our outcome variable was the mean number of dental caries experienced by 12-year-old children, indicated by decayed, missing, or filled permanent teeth (DMFT), because it reflects the population benefit of S-FMR. These data were obtained from records of school dental health checkups, which are required by law, conducted between 2006 and 2012.^33^ To consider dental caries experience before receiving S-FMR, mean numbers of decayed, missing, or filled primary teeth (dmft) of 3-year-old children were obtained from dental health checkups at local health centers, which are also required by law, conducted between 1997 and 2003.^34^ Dental caries experience of primary teeth could represent dental caries risk for permanent teeth in each prefecture because past caries experience is the best predictor of future dental caries onset in children.^35^

### S-FMR utilization

The S-FMR variable was the proportion of children in the prefecture who received S-FMR, obtained from the National Survey on School-based Fluoride Mouth-rinse Programs in Japan.^36^ Children aged 6–7 years are in the first grade of elementary school in Japan. Longer periods of S-FMR exposure are more beneficial to prevent dental caries.^21^ Because the proportion of S-FMR varied annually, we used the data between 2001 and 2007 for each birth cohort, which indicated the S-FMR proportion in each prefecture when children were aged 7 years. This survey was conducted biennially. Mean values of the results of 1 year prior or later were used for the years in which the survey was not conducted.

### Other variables

To consider other factors that possibly contribute to dental caries and caries-related inequalities between prefectures, we obtained the following variables from surveys conducted when children were 7 years old (between 2001 and 2007): 1) average consumption of fluoride toothpaste per capita in each prefecture, calculated by multiplying domestic utilization of fluoride toothpaste^37^ and average number of times any toothpaste was bought per capita in a year^38^; 2) dentist density (per 100 000 people)^39^; 3) average sugar consumption per capita in each prefecture^38^; and 4) mean annual income of each prefecture.^40^ The survey of dentist density was conducted biennially. Mean values of the results of one year prior or later were used for the years the survey was not conducted. We could not consider water fluoridation because it was not conducted in Japan. Since only aggregated open data were used, ethical approval was not needed.

### Analysis

We applied multivariable multilevel linear regression models to examine S-FMR’s contribution to decreasing dental caries experience and caries-related inequalities. For the current data set, every prefecture had seven birth cohorts. Therefore, the year was treated as level 1, nested within the prefecture as level 2. To determine the contribution of a 1% increment in the S-FMR coverage on dental caries and caries-related inequalities, S-FMR was treated as a continuous variable. Other variables except dentist density were also treated as continuous variables. Continuous variables were centered on the grand mean values. Dentist density was divided into quartile categories, as it was skewed.

We constructed five models, sequentially adding independent variables: a model to check whether there is a significant DMFT difference at 12 years old between prefectures with no explanation variable (model 1); a model to examine contributions of fluoride toothpaste dissemination, mean annual income, sugar consumption, and dentist density to the DMFT difference at 12 years old (model 2); a model to examine contribution of dental caries experience at 3 years old (model 3); a model to examine the contribution of S-FMR (model 4); and a model to examine whether S-FMR contributes to decreasing DMFT inequalities at 12 years old between prefectures, adding interaction terms of dental caries experience at 3 years old and S-FMR utilization (model 5). If model 5 shows that S-FMR is more effective in lowering DMFT for 12-year-olds in the prefectures with high dental caries experience at age 3, it indicates that S-FMR contributes to improving dental caries inequalities between prefectures. In addition, to estimate the modified S-FMR effects by dental caries experience at age 3, we assumed three situations: dental caries experience at age 3 was low (mean −1 standard deviation [SD]); middle (mean); or high (mean +1 SD).^41^ Then, the associations between S-FMR and DMFT at age 12 years in each situation were estimated. Model fitting was evaluated by Akaike’s information criterion (AIC) and the likelihood ratio test.

We also conducted sensitivity analysis because the S-FMR distribution was skewed. In the sensitivity analysis, we divided the S-FMR variable into deciles and treated it as a categorical variable. To evaluate the dose-response relationship of S-FMR on DMFT at age 12 years, *P*-values for trends were calculated. Interaction terms were not examined in the sensitivity analysis.

All analyses were conducted using the Stata 13.1 software package (Stata Corp LP, College Station, TX, USA).

## RESULTS

Table 1 shows descriptive statistics for the pooled data from seven birth cohorts in all 47 prefectures. Mean (SD) DMFT for 12-year-olds was 1.53 (0.48) and mean (SD) S-FMR utilization was 4.77 (7.49). S-FMR utilization was not significantly associated with dmft for 3-year-olds (eTable 2).

**Table 1.** Descriptive statistics of the number of decayed, missing, and filled permanent teeth for 12-year-olds and S-FMR utilization from seven birth cohorts in 47 prefectures (number of prefecture data = 329)

	mean (SD)	minimum	maximum	percentile		

				25th	50th	75th
DMFT for 12-year-olds	1.53 (0.48)	0.60	3.50	1.20	1.50	1.80
Fluoride toothpaste consumption^a^	1.37 (0.15)	0.96	1.73	1.26	1.38	1.48
Income, 10 000 USD^b^	2.74 (0.41)	1.99	4.82	2.45	2.71	2.95
Sugar consumption, kg^c^	26.91 (5.97)	14.73	58.17	22.29	26.01	30.49
Dentist density^d^	66.69 (14.20)	45.23	126.89	57.25	63.46	72.11
dmft at 3 years old^e^	1.82 (0.57)	0.78	3.62	1.37	1.73	2.20
S-FMR utilization, %^f^	4.77 (7.49)	0.00	50.45	0.70	1.75	6.15

DMFT, total number of decayed, missing, or filled permanent teeth; dmft, total number of decayed, missing, or filled primary teeth; SD, standard deviation; S-FMR, school-based fluoride mouth-rinse program.

^a^Average number of times buying fluoride toothpaste per year in each prefecture.

^b^Average annual income in each prefecture (1 USD = 100 JPY).

^c^Average sugar consumption per capita in each prefecture.

^d^Number of dentists per 100 000 residents in each prefecture.

^e^Average number of dmft per capita at age 3.

^f^Proportion of children who receive S-FMR in each prefecture.

**Figure 1.**
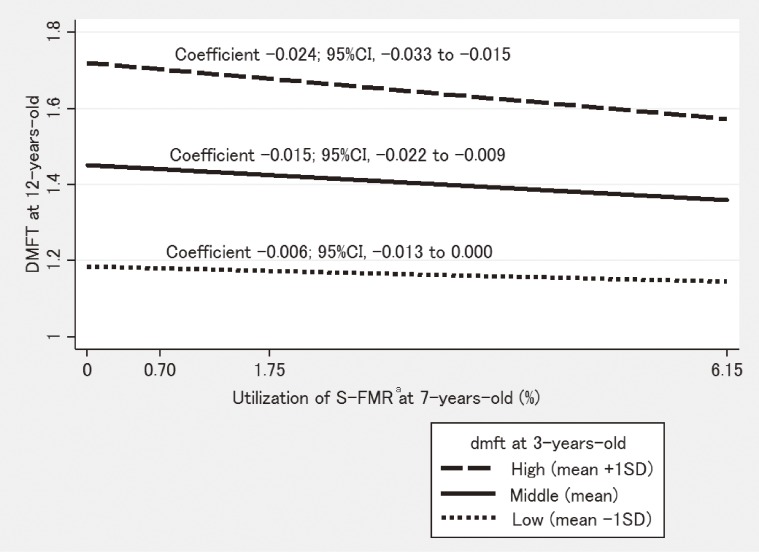
Effect modification of S-FMR by dental caries status at 3 years old CI, confidence interval; DMFT, total number of decayed, missing, or filled permanent teeth; dmft, total number of decayed, missing, or filled primary teeth; SD, standard deviation; S-FMR, school-based fluoride mouth-rinse programs. ^a^Proportion of children who receive S-FMR in each prefecture

Table 2 shows the results of multivariable multilevel linear regression analyses. There were significant prefecture- and year-level differences in DMFT for 12-year-olds: 70.4% of the variation occurred between prefectures and 29.6% of the variation occurred between years (model 1). Higher utilization of fluoride toothpaste, higher income, and higher dentist density were significantly associated with lower DMFT (model 2). Dental caries experience at 3 years old seemed to mediate the influence of these associations (model 3). However, even after considering these variables, prefecture-level differences remained significant (models 2 and 3). An increase of 1% in S-FMR utilization was significantly associated with 0.011 lower DMFT in 12-year-olds, even after considering other variables (model 4: coefficient −0.011; 95% confidence interval [CI], −0.018 to −0.005). In addition to the dental caries prevention effect, S-FMR seemed to reduce dental caries inequalities. S-FMR largely reduced the prefecture-level variance compared to other variables: 25.2% of the variance was explained by S-FMR in model 4, whereas 21.3% was explained by other variables in model 2. There was significant interaction between dental caries experience at age 3 and S-FMR utilization. S-FMR was more effective among the prefectures with high dental caries experience at age 3 (model 5: coefficient −0.015; 95% CI, −0.023 to −0.007). When dental caries experience at age 3 in the prefecture was low, middle, and high, an increase of 1% in S-FMR utilization was associated with 0.006, 0.015, and 0.024 lower DMFT, respectively, at age 12 years (Figure 1). Model fits were significantly improved when variables were added (Table 2).

**Table 2.** Association between school-based fluoride mouth-rinse program and the number of decayed, missing, and filled permanent teeth for 12-year-olds at the prefectural level: a 7-year birth cohort multilevel analysis

	model 1	model 2	model 3	model 4	model 5
	Coefficient (95% CI)	Coefficient (95% CI)	Coefficient (95% CI)	Coefficient (95% CI)	Coefficient (95% CI)
Fixed part					
Intercept	1.526 (1.407, 1.646)	1.796 (1.652, 1.940)	1.652 (1.527, 1.777)	1.705 (1.588, 1.822)	1.636 (1.527, 1.745)
Fluoride toothpaste consumption^a^		−0.862 (−1.053, −0.670)	−0.187 (−0.359, −0.016)	−0.185 (−0.356, −0.015)	−0.214 (−0.382, −0.046)
Income, 10 000 USD^b^		−0.356 (−0.551, −0.160)	−0.091 (−0.256, 0.074)	−0.081 (−0.236, 0.075)	−0.098 (−0.249, 0.053)
Sugar consumption, kg^c^		0.005 (−0.002, 0.012)	0.001 (−0.004, 0.006)	0.001 (−0.005, 0.006)	0.001 (−0.004, 0.007)
Dentist density^d^					
<57.21		reference	reference	reference	reference
57.21–63.46		−0.252 (−0.362, −0.141)	−0.123 (−0.210, −0.035)	−0.130 (−0.217, −0.044)	−0.122 (−0.206, −0.037)
63.47–72.16		−0.379 (−0.526, −0.233)	−0.198 (−0.316, −0.080)	−0.182 (−0.297, −0.067)	−0.162 (−0.275, −0.049)
>72.17		−0.446 (−0.641, −0.251)	−0.183 (−0.345, −0.021)	−0.185 (−0.339, −0.030)	−0.160 (−0.310, −0.010)
dmft at 3 years old^e^			0.623 (0.540, 0.705)	0.569 (0.484, 0.653)	0.539 (0.456, 0.623)
S-FMR utilization, %^f^				−0.011 (−0.018, −0.005)	−0.015 (−0.022, −0.009)
dmft at 3 years old # S-FMR utilization, %					−0.015 (−0.023, −0.007)
Random part					
Prefecture-level variance (SE)	0.164 (0.036)***	0.129 (0.028)***	0.107 (0.024)***	0.080 (0.018)***	0.071 (0.016)***
Year-level variance (SE)	0.069 (0.006)***	0.042 (0.004)***	0.024 (0.002)***	0.024 (0.002)***	0.024 (0.002)***
AIC	195.720	55.980	−108.090	−116.680	−128.190
*P*-value for likelihood ratio test^g^	—	<0.001	<0.001	0.001	<0.001

*P < 0.05; **P < 0.01; ***P < 0.001.

AIC, Akaike’s information criterion; CI, confidence interval; DMFT, total number of decayed, missing, or filled permanent teeth; dmft, total number of decayed, missing, or filled primary teeth; SE, standard error; S-FMR, school-based fluoride mouth-rinse programs.

Coefficient represents the degree of decline in DMFT for 12-year-olds by one percent increment of S-FMR.

^a^Average number of times buying fluoride toothpaste per year in each prefecture.

^b^Average annual income in each prefecture (1 USD = 100 JPY).

^c^Average sugar consumption per capita in each prefecture.

^d^Number of dentists per 100 000 residents in each prefecture.

^e^Average number of dmft per capita at age 3 years.

^f^Proportion of children who receive S-FMR in each prefecture.

^g^Compared to one-prior model.

**Figure 2.**
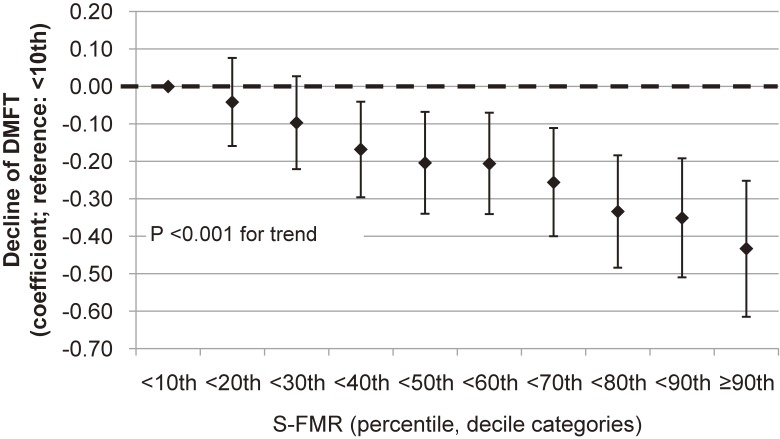
Decline of the number of decayed, missing, and filled permanent teeth for 12-year-olds by decile categories of S-FMR. Vertical bars indicate 95% confidence intervals. DMFT, total number of decayed, missing, or filled permanent teeth; S-FMR, school-based fluoride mouth-rinse program.

Sensitivity analysis using S-FMR deciles showed that over the 40th percentile of S-FMR utilization showed significantly lower DMFT at age 12 years compared to that at under the 10th percentile of the S-FMR utilization group (Figure 2). There was a dose-response relationship between S-FMR and DMFT for 12-year-olds (*P* < 0.001 for trend).

## DISCUSSION

This study showed that each 1% increase in S-FMR utilization was significantly associated with 0.011 lower DMFT for 12-year-olds. In Japan, there were 1 314 403 children aged 12 years in 2000.^42^ Their estimated total number of DMFT was 1 616 716 because average DMFT for 12-year-olds was 1.23 in 2000. Therefore, a 1% increment of S-FMR utilization corresponds to 13 144 children aged 12 years who received S-FMR when they were 6 years old; from the coefficients of S-FMR in model 4 (−0.011), the calculated number of DMFT decline was 14 458 (0.89% of the total number of DMFT for 12-year-olds in 2000) in this hypothetical situation. Thus, S-FMR would have large benefits for population dental health. However, this estimated reduction in the number of caries needs careful interpretation because this is an ecological study and the effect of S-FMR differs by children’s risk factors and other characteristics, such as caries risk and fluoride utilization. S-FMR contributed to prefecture-level DMFT variance even in the present situation where fluoride toothpaste is widely used. This result was robust in our sensitivity analyses. The association between S-FMR and DMFT for 12-year-olds was modified by dental caries experience at age 3 years (dmft at 3 years old) in the prefectures. S-FMR had more beneficial associations with DMFT for 12-year-olds when mean numbers of dental caries experienced at age 3 years in the prefecture were high. Thus, S-FMR seemed to decrease inequalities in dental caries between prefectures.

Our study examined the effect of FMR under actual social conditions, rather than in an experimental or randomized interventional context. Randomization has many advantages for estimating intervention effects.^43^ However, the external validity of randomized trials may be limited in real-world settings. Participants who are included and followed in randomized intervention studies might differ from the general population.^32^^,^^43^^,^^44^ Additionally, according to the “inverse care law” concept first discussed by Hart JT in 1971, interventions that depend on individual behavior are more effective among low-risk than high-risk populations.^13^^,^^14^^,^^45^ Although randomized controlled trials showed little additional effects of fluoride mouth-rinse beyond fluoride toothpaste alone on dental caries prevention,^29^ our study showed a beneficial effect of S-FMR in a society where fluoride toothpaste is widely used. One possible reason for this finding is that the utilization of fluoride toothpaste among children is affected by their socioeconomic status.^46^ Utilization of fluoride toothpaste is likely low in children of low socioeconomic status.^27^ Unfortunately, such children tend to be at high risk for dental caries.^18^ In contrast, S-FMR is an intervention that changes school environment as a means of improving schoolchildren’s dental health. Therefore, its advantages are easily conferred to children of low socioeconomic status. This may partly account for the results of our study.

Health interventions can be divided into two types: upstream interventions and downstream interventions.^13^^,^^47^ Upstream interventions (eg, water fluoridation and S-FMR) change health determinants at the social, political, and life circumstance levels and decrease health inequalities.^3^^,^^18^^,^^25^^,^^48^^,^^49^ Downstream interventions (eg, health education to change health behavior) alter health determinants at the individual level and increase health inequalities because such interventions are often more effective for low-risk than high-risk individuals.^13^^,^^43^ The use of fluoride toothpaste at home is a downstream intervention, as it depends on individual health behavior and socioeconomic status. In some countries, toothpaste is considered cosmetic, and many families are unable to obtain it because of its high cost.^3^ In addition, an intervention that provided free fluoride toothpaste for children in England did not decrease dental caries inequalities related to deprivation.^27^ On the other hand, some upstream interventions have been shown to reduce dental health inequalities. Several reviews have indicated that caries inequalities across social classes have decreased in countries where water fluoridation has been implemented.^18^^,^^48^^,^^50^ Although water fluoridation is one of the most cost-effective public health strategies,^3^^,^^15^ it is unfortunately not conducted in Japan. Results of the present study showed geographical and socioeconomic inequalities in dental caries. Therefore, upstream interventions are also needed in Japan. S-FMR, an intervention that changes school environments to improve schoolchildren’s health, is a relatively upstream public health intervention conducted in Japan.

This study suggests that S-FMR as a population strategy could be effective for reducing dental caries and caries-related geographical inequalities, even in areas/countries where fluoride toothpaste is widely used and dental caries prevalence has been declining. Theoretically, it is possible that S-FMR is not randomly distributed because prefectures or schools with high dmft for 3-year-olds might be more likely to implement S-FMR. In such situations, S-FMR would work as a geographical targeting approach and contribute to reducing inequality in dental caries among prefectures. In this study, S-FMR was not significantly associated with dmft for 3-year-olds; prefectures with higher caries levels did not tend to conduct S-FMR. However, S-FMR reduced inequalities in DMFT for 12-year-olds; thus, it worked as a geographical targeting approach. The possible reason for reduction of inequalities by S-FMR is that children in prefectures with lower caries prevalence already tended to use fluoride; therefore, the effects of S-FMR on caries rates and inequalities are attenuated.^28^ In contrast, usage of fluoride in prefectures with higher prevalence of caries is not prevalent; therefore, additional effects of S-FMR are greater and inequalities are reduced. Figure 1 supports this hypothesis of the different effectiveness of S-FMR by prefecture caries level. School-based programs have a potential benefit for dental public health because of their wide population reach.^21^ Therefore, more widespread S-FMR implementation is recommended. The importance of “proportionate universalism” was proposed to reduce health inequalities. Proportionate universalism refers to interventions that improve the health of high risk groups more than that of low risk groups.^51^ S-FMR seems to work via proportionate universalism to reduce health inequalities.

The strength of this study was that we investigated the impact of S-FMR on population dental health and caries-related geographical inequalities using multilevel analyses of longitudinal data. We also considered other possible factors that could explain the geographical inequalities in dental caries. The present approach enabled us to examine the impact of S-FMR under actual social conditions. On the other hand, this study has several limitations. First, this was a prefecture-based ecological study. In general, ecological study designs do not allow causal inferences because an association observed between variables on an aggregated level does not necessarily represent the association at an individual level (the “ecological fallacy”).^31^ However, we intended to investigate the population effect of S-FMR on dental health and its contribution to inequalities in dental caries between prefectures. Ecological studies are appropriate for implications of prevention or intervention implications at a population level.^31^ In fact, ecological studies are frequently used in geographical epidemiology.^30^ Additionally, the ecological fallacy can be avoided when individual-level causation has been shown in previous studies.^32^ Thus, ecological fallacy was less of a concern in the present study because the effect of fluoride on dental caries prevention has been demonstrated in many previous studies.^50^^,^^52^ Second, some schoolchildren might have received fissure sealant on their permanent teeth, but we did not have information on fissure sealant. However, we believe that this bias did not seriously affect the result because no fissure sealant programs were conducted at the prefecture level. Basically, Japanese children who receive fissure sealant in dental clinics do so under on individual basis, not at school. A significant association between S-FMR and fissure sealant coverage is therefore unlikely. Thus, it is improbable that the absence of data on fissure sealant resulted in overestimation of the effect of S-FMR in this study. Third, the S-FMR data in the present study included FMR utilization in preschool, kindergarten, elementary school, junior high school, and schools for special-needs education. In Japan, children in junior high school and some children in schools for special-needs education are 13 years or older. This could cause some bias because S-FMR for them would not have had causal effects on the outcome of the present study (ie, DMFT among 12-year-olds). However, S-FMR utilization excluding that in junior high schools and schools for special-needs education in each prefecture, which was available only for 2010,^23^ and S-FMR utilization in the prefecture were highly correlated (*r* = 0.99, *P* < 0.001). Therefore, the results of this study were reliable. Fourth, we obtained information on dental caries from mandatory dental health check-up surveys, in which many dentists in Japan examine children’s dental status. Inter-validity between the dentists in this survey has not been evaluated, and might have produced a degree of bias. However, the dentists employed universal caries diagnosis criteria. Nonetheless, some dentists may underestimate children’s dental caries, whereas others may overestimate it. Such “non-differential misclassification” bias would lead to underestimation of the results. We observed significant associations even with this potential bias, and therefore consider that this bias did not seriously affect our results. Finally, we followed birth cohorts at the prefecture level. Some individuals might have moved to other prefectures during the follow-up, which might have caused some bias. However, this bias would also be “non-differential misclassification” because moving to other prefectures would not be associated with S-FMR utilization or dental caries. Therefore, we believe the associations we observed in the present study are robust.

In conclusion, this study showed that S-FMR was associated with low dental caries experience and decreasing caries-related inequalities between prefectures, even in a country where fluoride toothpaste is widely used and dental caries prevalence has been decreasing.

## ONLINE ONLY MATERIALS

eFigure 1. Timeline of surveys from which variables were obtained.

eTable 1. Birth year and linked survey year. We merged surveys for each birth cohort by age (eg, the survey on dental caries status at 3 years old in 1997; the 2001 surveys on S-FMR utilization, annual income in prefectures, dentist density, fluoride toothpaste consumption, and sugar consumption; and the survey on DMFT for 12-year-olds in 2006, were merged o the birth cohort born in 1994). Each prefecture had 7 years of data (children born between 1994 and 2000).

eTable 2. Associations between the number of decayed, missing, and filled primary teeth at 3 years old and each explanatory variable, stratified by birth year.

Abstract in Japanese.

## References

[1] Williams DM Global oral health inequalities: the research agenda. J Dent Res. 2011;90:549–51. 10.1177/0022034511402210 21490241

[2] Petersen PE Oral Health. International Encyclopedia of Public Health. 2008;1:677–85.

[3] Blas E, Kurup AS; World Health Organization. Equity, social determinants, and public health programmes. Geneva, Switzerland: World Health Organization; 2010.

[4] Marcenes W, Kassebaum NJ, Bernabé E, Flaxman A, Naghavi M, Lopez A, Global burden of oral conditions in 1990–2010: a systematic analysis. J Dent Res. 2013;92:592–7. 10.1177/0022034513490168 23720570 PMC4484374

[5] Petersen PE The World Oral Health Report 2003: continuous improvement of oral health in the 21st century—the approach of the WHO Global Oral Health Programme. Community Dent Oral Epidemiol. 2003;31 Suppl 1:3–23. 10.1046/j..2003.com122.x 15015736

[6] Aida J, Ando Y, Oosaka M, Niimi K, Morita M Contributions of social context to inequality in dental caries: a multilevel analysis of Japanese 3-year-old children. Community Dent Oral Epidemiol. 2008;36:149–56. 10.1111/j.1600-0528.2007.00380.x 18333879

[7] Tanaka K, Miyake Y, Sasaki S, Hirota Y Socioeconomic status and risk of dental caries in Japanese preschool children: the Osaka Maternal and child health study. J Public Health Dent. 2013;73:217–23. 10.1111/jphd.12016 23560765

[8] Lee JY, Divaris K The ethical imperative of addressing oral health disparities: a unifying framework. J Dent Res. 2014;93:224–30. 10.1177/0022034513511821 24189268 PMC3929974

[9] Do LG Distribution of caries in children: variations between and within populations. J Dent Res. 2012;91:536–43. 10.1177/0022034511434355 22223436

[10] Public Health England. National Dental Epidemiology Programme for England: oral health survey of five-year-old children 2012. London: PHE; 2013.

[11] Aida J, Ando Y, Aoyama H, Tango T, Morita M An ecological study on the association of public dental health activities and sociodemographic characteristics with caries prevalence in Japanese 3-year-old children. Caries Res. 2006;40:466–72. 10.1159/000095644 17063016

[12] Ministry of Health Labour and Welfare. Estimates of National Medical Care Expenditure; 2011.

[13] Lorenc T, Petticrew M, Welch V, Tugwell P What types of interventions generate inequalities? Evidence from systematic reviews. J Epidemiol Community Health. 2013;67:190–3. 10.1136/jech-2012-201257 22875078

[14] Schou L, Wight C Does dental health education affect inequalities in dental health? Community Dent Health. 1994;11:97–100. 8044719

[15] Petersen PE, Kwan S Equity, social determinants and public health programmes—the case of oral health. Community Dent Oral Epidemiol. 2011;39:481–7. 10.1111/j.1600-0528.2011.00623.x 21623864

[16] Woodward A, Kawachi I Why reduce health inequalities? J Epidemiol Community Health. 2000;54:923–9. 10.1136/jech.54.12.923 11076989 PMC1731601

[17] Rose GA. Rose’s strategy of preventive medicine: the complete original text. New York: Oxford University Press; 2008.

[18] Burt BA Fluoridation and social equity. J Public Health Dent. 2002;62:195–200. 10.1111/j.1752-7325.2002.tb03445.x 12474623

[19] Japanese Society for Oral Health Statement for society without dental caries. J Dent Hlth. 2013;63:400–11 [in Japanese].

[20] Takiguchi T Dental health in 21st century. Koshu eisei. 2001;65:510–3 [in Japanese].

[21] Divaris K, Rozier RG, King RS Effectiveness of a school-based fluoride mouth-rinse program. J Dent Res. 2012;91:282–7. 10.1177/0022034511433505 22202124

[22] Ministry of Health Labour and Welfare. Guideline on fluoride mouthrinsing; 2003.

[23] Komiyama K, Kimoto K, Taura K, Sakai O National survey on school-based fluoride mouth-rinsing programme in Japan: regional spread conditions from preschool to junior high school in 2010. Int Dent J. 2014;64:127–37. 10.1111/idj.12068 24256345 PMC4255315

[24] Burt BA Concepts of risk in dental public health. Community Dent Oral Epidemiol. 2005;33:240–7. 10.1111/j.1600-0528.2005.00231.x 16008630

[25] Daly B. Essential dental public health. 1st ed. New York: Oxford University Press; 2002.

[26] Levin KA, Jones CM, Wight C, Valentine C, Topping GV, Naysmith R Fluoride rinsing and dental health inequalities in 11-year-old children: an evaluation of a supervised school-based fluoride rinsing programme in Edinburgh. Community Dent Oral Epidemiol. 2009;37:19–26. 10.1111/j.1600-0528.2008.00445.x 19046333

[27] Ellwood RP, Davies GM, Worthington HV, Blinkhorn AS, Taylor GO, Davies RM Relationship between area deprivation and the anticaries benefit of an oral health programme providing free fluoride toothpaste to young children. Community Dent Oral Epidemiol. 2004;32:159–65. 10.1111/j.1600-0528.2004.00150.x 15151685

[28] Marinho VC, Higgins JP, Sheiham A, Logan S Combinations of topical fluoride (toothpastes, mouth-rinses, gels, varnishes) versus single topical fluoride for preventing dental caries in children and adolescents. Cochrane Database Syst Rev. 2004;(1):CD002781. 14973992 10.1002/14651858.CD002781.pub2 PMC6999808

[29] Marinho VC, Higgins JP, Logan S, Sheiham A Topical fluoride (toothpastes, mouth-rinses, gels or varnishes) for preventing dental caries in children and adolescents. Cochrane Database Syst Rev. 2003;(4):CD002782. 14583954 10.1002/14651858.CD002782 PMC6999805

[30] Elliott P, Cuzick J, English D, Stern R. Geographical and environmental epidemiology: methods for small-area studies. Oxford; New York. Published on behalf of the World Health Organization Regional Office for Europe by Oxford University Press; 1996.

[31] Szklo M, Nieto FJ. Epidemiology: beyond the basics. 2nd ed. Sudbury, Mass: Jones and Bartlett Publishers; 2007.

[32] Raymond G, Stephen D, Dana F, John E, John B. Medical Epidemiology. 4th ed. New York: Lange Medical Books; 2004.

[33] Ministry of Health Labour and Welfare. School Health Statistics Research; 2006–2012.

[34] National Institute of Public Health. Dental health checkups for 3-year-olds; 1997–2003.

[35] Mejàre I, Axelsson S, Dahlén G, Espelid I, Norlund A, Tranæus S, Caries risk assessment. A systematic review. Acta Odontol Scand. 2014;72:81–91. 10.3109/00016357.2013.822548 23998481

[36] Non-profit Japanese Conference on the Promotion of the Use of Fluoride in Caries Prevention. National Survey on the School-based Fluoride Mouthrinsing Program in Japan; 2001–2007.

[37] Non-profit Japanese Conference on the Promotion of the Use of Floride in Caries Prevention. List of Fluoride Products in Japan. 9th ed. Tokyo, Japan: Oral Health Association of Japan; 2010.

[38] Statistics Bureau Ministry of Internal Affairs and Communications. Family income and expenditure survey; 2001–2007.

[39] Ministry of Health Labour and Welfare. Survey of Physicians, Dentists and Pharmacists. 2001–2007.

[40] Cabinet Office. Prefectural accounts. 2001–2007.

[41] Cohen J. Applied multiple regression/correlation analysis for the behavioral sciences. 3rd ed. Mahwah, N.J.: L. Erlbaum Associates; 2003.

[42] Ministry of Internal Affairs and Communications. Population census; 2000.

[43] Katz MH. Evaluating Clinical and Public Health Interventions: A Practical Guide to Study Design and Statistics. 1st ed. New York: Cambridge University Press; 2010.

[44] Egger M, Schneider M, Davey Smith G Meta-analysis Spurious precision? Meta-analysis of observational studies. BMJ. 1998;316(7125):140–4. 10.1136/bmj.316.7125.140 9462324 PMC2665367

[45] Hart JT The inverse care law. Lancet. 1971;1(7696):405–12. 10.1016/S0140-6736(71)92410-X 4100731

[46] Jürgensen N, Petersen PE Oral health behaviour of urban and semi-urban schoolchildren in the Lao PDR. Community Dent Health. 2011;28:280–5. 22320066

[47] Watt RG From victim blaming to upstream action: tackling the social determinants of oral health inequalities. Community Dent Oral Epidemiol. 2007;35:1–11. 10.1111/j.1600-0528.2007.00348.x 17244132

[48] Riley JC, Lennon MA, Ellwood RP The effect of water fluoridation and social inequalities on dental caries in 5-year-old children. Int J Epidemiol. 1999;28:300–5. 10.1093/ije/28.2.300 10342695

[49] Babones SJ. How and why do interventions that increase health overall widen inequalities within populations. Policy Press Scholarship Online; 2009.

[50] Fejerskov O, Kidd E. Dental Caries: The Disease and Its Clinical Management. 2nd ed. Munksgaard. Wiley-Blackwell; 2008.

[51] Marmot M, Atkinson T, Bell J, Black C, Broadfoot P, Cumberlege J, et al. Fair Society, Healthy Lives: The Marmot Review, strategic review of health inequalities in England post—2010. In Marmot M (Ed.); 2010.

[52] Marinho VC, Higgins JP, Sheiham A, Logan S Fluoride toothpastes for preventing dental caries in children and adolescents. Cochrane Database Syst Rev. 2003;CD002278. 12535435 10.1002/14651858.CD002278 PMC8439270

