# Supplementary material for: School-Based Fluoride Mouth-Rinse Program Dissemination Associated With Decreasing Dental Caries Inequalities Between Japanese Prefectures: An Ecological Study
Source: J Epidemiol. 2016 Nov 5;26(11):563–71. doi: 10.2188/jea.JE20150255 (PMC5083319; doi:10.2188/jea.JE20150255)
Supplement: eFigure 1. [file je-26-563-s001.pdf]

eFigure 1. Timeline of surveys from which variables were obtained

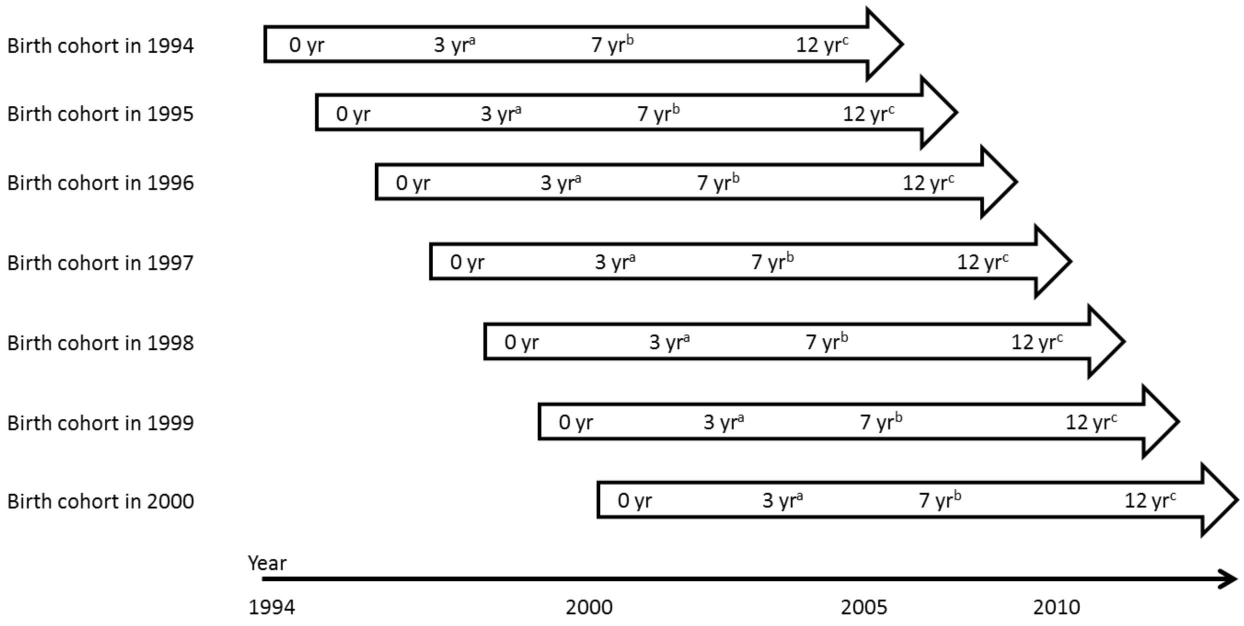

yr, years old.

All variables were aggregated to prefecture-level.

[a] Decayed, missing, or filled primary teeth were obtained.

[b] Proportion of children who receive school-based fluoride mouth-rinse programs, average number of times buying fluoride toothpaste, average annual income, average sugar consumption, and dentist density were obtained.

[c] Decayed, missing, or filled permanent teeth were obtained.
