# Supplementary material for: School-Based Fluoride Mouth-Rinse Program Dissemination Associated With Decreasing Dental Caries Inequalities Between Japanese Prefectures: An Ecological Study
Source: J Epidemiol. 2016 Nov 5;26(11):563–71. doi: 10.2188/jea.JE20150255 (PMC5083319; doi:10.2188/jea.JE20150255)
Supplement: eTable 1. [file je-26-563-s002.pdf]

eTable 1. Birth year and linked survey year. We merged surveys for each birth cohort by age (e.g., the survey on dental caries status at 3 years old in 1997; the 2001 surveys on S-FMR utilization, annual income in prefectures, dentist density, fluoride toothpaste consumption, and sugar consumption; and the survey on DMFT for 12-year-olds in 2006, were merged to the birth cohort born in 1994). Each prefecture had 7 years of data (children born between 1994 and 2000).

Prefecture[a]	Birth year	dmft for 3-years old[b]	Utilization of S-FMR[c]	Income[d]	Density of dentists[e]	Fluoride toothpaste consumption[f]	Sugar consumption[g]	DMFT for 12-years old[h]
Tokyo	1994	1997	2001	2001	2001	2001	2001	2006
Tokyo	1995	1998	2002	2002	2002	2002	2002	2007
Tokyo	1996	1999	2003	2003	2003	2003	2003	2008
Tokyo	1997	2000	2004	2004	2004	2004	2004	2009
Tokyo	1998	2001	2005	2005	2005	2005	2005	2010
Tokyo	1999	2002	2006	2006	2006	2006	2006	2011
Tokyo	2000	2003	2007	2007	2007	2007	2007	2012
Hokkaido	1994	1997	2001	2001	2001	2001	2001	2006
Hokkaido	1995	1998	2002	2002	2002	2002	2002	2007
Hokkaido	1996	1999	2003	2003	2003	2003	2003	2008
Hokkaido	1997	2000	2004	2004	2004	2004	2004	2009
Hokkaido	1998	2001	2005	2005	2005	2005	2005	2010
Hokkaido	1999	2002	2006	2006	2006	2006	2006	2011
Hokkaido	2000	2003	2007	2007	2007	2007	2007	2012
Aomori	1994	1997	2001	2001	2001	2001	2001	2006
Aomori	1995	1998	2002	2002	2002	2002	2002	2007
Aomori	1996	1999	2003	2003	2003	2003	2003	2008

DMFT, number of decayed, missing, and filled permanent teeth; S-FMR, school-based fluoride mouth-rinse programs

[a] 47 prefectures (Tokyo, Hokkaido, Aomori, Iwate, Miyagi, Akita, Yamagata, Fukushima, Ibaraki, Tochigi, Gunma, Saitama, Chiba, Kanagawa, Niigata, Toyama, Ishikawa, Fukui, Yamanashi, Nagano, Gifu, Shizuoka, Aichi, Mie, Shiga, Kyoto, Osaka, Hyogo, Nara, Wakayama, Tottori, Shimane, Okayama, Hiroshima, Yamaguchi,

Tokushima, Kagawa, Ehime, Kochi, Fukuoka, Saga, Nagasaki, Kumamoto, Oita, Miyazaki, Kagoshima, and Okinawa) had 7 birth cohorts (children born in 1994, 1995, 1996, 1997, 1998, 1999, and 2000).

[b] Average number of decayed, missing, or filled primary teeth at age 3

[c] Proportion of children who receive S-FMR in each prefecture

[d] Average annual income in each prefecture

[e] Number of dentists per 100,000 residents in each prefecture

[f] Average number of times buying fluoride toothpaste in each prefecture

[g] Average sugar consumption per capita in each prefecture

[h] Average number of decayed, missing, or filled permanent teeth at age 12
