# Supplementary material for: School-Based Fluoride Mouth-Rinse Program Dissemination Associated With Decreasing Dental Caries Inequalities Between Japanese Prefectures: An Ecological Study
Source: J Epidemiol. 2016 Nov 5;26(11):563–71. doi: 10.2188/jea.JE20150255 (PMC5083319; doi:10.2188/jea.JE20150255)
Supplement: eTable 2. [file je-26-563-s003.pdf]

eTable 2. Associations between the number of decayed, missing, and filled primary teeth at 3 years old and each explanatory variable, stratified by birth year

	Spearman's rho						
Birth year	1994	1995	1996	1997	1998	1999	2000
Fluoride toothpaste consumption[a]	-0.17	0.15	-0.08	-0.06	0.04	0.15	0.01
Income, 10,000 USD[b]	-0.65***	-0.63***	-0.59***	-0.65***	-0.67***	-0.69***	-0.60***
Sugar consumption, kg[c]	0.09	0.12	0.02	0.04	0.29*	0.40**	0.37*
Dentist density[d]	-0.26	-0.30*	-0.27	-0.25	-0.24	-0.26	-0.28
S-FMR utilization, %[e]	0.19	0.10	0.13	0.12	0.08	0.15	0.06

S-FMR, school-based fluoride mouth-rinse program.

* $p<0.05$

** $p<0.01$

*** $p<0.001$

[a] Average number of times buying fluoride toothpaste per year in each prefecture

[b] Average annual income in each prefecture (1 USD = 100 JPY)

[c] Average sugar consumption per capita in each prefecture

[d] Number of dentists per 100,000 residents in each prefecture

[e] Proportion of children who receive S-FMR in each prefecture
