# Supplementary material for: School-Based Fluoride Mouth-Rinse Program Dissemination Associated With Decreasing Dental Caries Inequalities Between Japanese Prefectures: An Ecological Study
Source: J Epidemiol. 2016 Nov 5;26(11):563–71. doi: 10.2188/jea.JE20150255 (PMC5083319; doi:10.2188/jea.JE20150255)
Supplement: Abstract in Japanese. [file je-26-563-s004.pdf]

小学校などでの集団フッ化物洗口と、むし歯の健康格差の縮小の関連：地域相関研究

松山　祐輔[1]、相田　潤[1]、田浦　勝彦[2]、木本　一成[3]、安藤　雄一[4]、青山　旬[5]、森田　学[6]、伊藤　奏[7]、
小山　史穂子[1]、長谷　晃広[1]、坪谷　透[1,8]、小坂　健[1]

1 東北大学大学院歯学研究科　国際歯科保健学分野、仙台、日本
2 NPO 法人日本フッ化物むし歯予防協会、瑞穂、岐阜、日本
3 神奈川歯科大学大学院歯学研究科　口腔科学講座　口腔衛生学分野、横須賀、神奈川、日本
4 国立保健医療科学院、生涯健康研究部、和光、埼玉、日本
5 栃木県立衛生福祉大学校歯科技術学部、宇都宮、日本
6 岡山大学大学院医歯薬学総合研究科予防歯科学分野、岡山、日本
7 埼玉県立大学　保健医療福祉学部　健康開発学科　口腔保健科学専攻、越谷、埼玉、日本
8 ハーバード・チャン公衆衛生大学院　社会行動科学学部　ボストン、マサチューセッツ、米国

背景：フッ化物配合歯磨剤が普及し、むし歯（う蝕）が減少している国でも、むし歯の健康格差は個人や社会の大きな負担となっている。保育園・幼稚園・小中学校・特別支援学校で実施される“学校フッ化物洗口（集団フッ化物洗口）”は、むし歯予防の優れたポピュレーションアプローチであり、むし歯の健康格差を縮小させている可能性がある。本研究は日本における学校フッ化物洗口と 12 歳児 1 人平均永久歯むし歯経験歯数（Decayed, Missing, or Filled permanent Teeth Index, DMFT 指数）の関連およびその都道府県格差との関連を明らかにすることを目的とした。

方法：1994-2000 年に生まれた子どもに関する、複数年度の都道府県レベルの集計データを使用し、地域相関研究をした。都道府県をレベル 2、出生年度をレベル 1 とした 2 レベルのマルチレベル線形回帰分析により、学校フッ化物洗口を受けている児童の割合（学校フッ化物洗口普及割合）と 12 歳児 DMFT 指数（平均永久歯むし歯経験歯数）の関連を検討した。共変量は 3 歳児 1 人平均乳歯むし歯経験歯数、歯科医師密度、都道府県の社会経済状況などとした。

結果：都道府県別の学校フッ化物洗口普及割合が高いことと 12 歳児 DMFT 指数が小さいことは有意に関連していた（B = -0.011; 95%信頼区間, -0.018 to -0.005）。他の変数を考慮してもなお、学校フッ化物洗口普及割合は 12 歳児 DMFT 指数の都道府県レベル分散を 25.2%説明した。学校フッ化物洗口普及割合と 3 歳児乳歯むし歯経験歯数の間に有意な交互作用がみられ、学校フッ化物洗口普及割合と 12 歳児 DMFT 指数の負の関連は、3 歳児乳歯むし歯経験歯数が多い都道府県で大きかった。

結論：学校フッ化物洗口普及割合が高いことは、12 歳児 DMFT 指数が小さいことと有意に関連していた。子どもの家庭環境や社会経済状況によらずに予防効果を受けることができる学校フッ化物洗口は、比例的普遍アプローチとしてはたらき、むし歯の健康格差を縮小していた。
